# Immune checkpoint inhibitors in large cell neuroendocrine carcinoma: current status

**DOI:** 10.18632/oncotarget.24553

**Published:** 2018-02-22

**Authors:** Aman Chauhan, Susanne M. Arnold, Jill Kolesar, Hala Elnakat Thomas, Mark Evers, Lowell Anthony

**Affiliations:** ^1^ Markey Cancer Center, University of Kentucky, Lexington, KY, USA; ^2^ University of Cincinnati, Cincinnati, OH, USA

**Keywords:** large cell neuroendocrine carcinoma, high grade neuroendocrine carcinoma, immune checkpoint inhibitors

## Abstract

**Introduction:**

Large cell neuroendocrine carcinomas (LCNEC) are a group of rare high grade neuroendocrine tumors that often behave clinically like small cell carcinoma (SCLC) and are treated as such. No major advancement in the management of these tumors has occurred in the last 30 years.

**Methods:**

We present a case series of three cases from Markey Cancer center along with a review of 13 published cases in the literature wherein immune-checkpoint inhibitors were utilized in the management of LCNEC.

**Results:**

Immune-checkpoint inhibitors might have clinical activity in LCNEC.

**Conclusion:**

Role of immune-checkpoint inhibitors should be explored in prospective LCNEC clinical trials. We summarize current evidence regarding use of immune checkpoint inhibitors in the treatment of LCNEC.

## INTRODUCTION

Large cell neuroendocrine carcinomas (LCNEC) are a group of rare high grade neuroendocrine tumors that often behave clinically like small cell carcinoma (SCLC) and are treated as such. These tumors can arise anywhere in the body, but pulmonary LCNEC is by far the most common. Patients usually present with extensive disease and have a poor prognosis. Platinum-based chemotherapy is often the treatment of choice, similar to SCLC, but response is short-lived. No standard second line treatment exists. Various combinations of taxane- or irinotecan-based chemotherapies have yielded poor response rates. Clinical trials are few and are difficult to conduct due to rarity of the disease. No major advancement in management of these tumors has occurred in the last 30 years. We summarize current evidence regarding immune checkpoint inhibitors in the treatment of LCNEC.

## RESULTS

To our knowledge, there are only two published case reports and one poster presentation that retrospectively reviewed the efficacy of immune checkpoint inhibitors in LCNEC. We would like to add our single center clinical experience with immune checkpoint inhibitors (*n* = 3) and also summarize the existing published clinical data to date.

Levra *et al*. presented their data on use of immune checkpoint inhibitors in pulmonary LCNEC at the IASLC 18th World Conference on Lung Cancer in 2017. Ten patients were treated with immune checkpoint inhibitors (9 with nivolumab and 1 with pembrolizumab). Six of the ten showed a partial response and one demonstrated stable disease. Median progression free survival was reported as 57 weeks and the median number of doses of immune checkpoint inhibitor therapy received was 16 [1].

Daido *et al*. reported 2 cases of LCNEC who received nivolumab as third and sixth line of salvage therapy for progressive metastatic disease. The authors reported a radiological response to immune checkpoint inhibitor therapy but the degree and duration of response was not presented [2].

Wang *et al*. reported a single case of pulmonary LCNEC in 2017 with an exceptional response to a first dose of pembrolizumab. The patient was continuing treatment at the time of publication of the case study so the duration of response cannot be determined [3].

Table 1 describes 3 cases of LCNEC managed at the University of Kentucky with ongoing durable response to immune checkpoint inhibitor therapy.

**Table 1:** LCNEC patients treated with immune checkpoint inhibitors at Markey Cancer Center, University of Kentucky

Patient	Prior treatment	Current treatment	Response
80 Y/O F with metastatic gastric LCNEC	6 cycles of cisplatin and etoposide. Disease progression in liver three months after platinum doublet completion.	Second line, off label nivolumab q 2 weeks for past 6 months and continuing.	Clinical and radiological response. Stable hepatic metastatic disease.
57 Y/O with metastatic LCNEC of lung with brain metastasis	Resection of brain metastasis followed by radiation, carboplatin and etoposide X 4 cycles, intolerance to further platinum doublet. Switched to maintenance pemetrexed X 21 cycles, developed toxicity to pemetrexed. Switched to off label nivolumab.	Nivolumab discontinued post 4 doses due to lack of measurable radiological disease. Currently on observation.	Complete response. Off therapy for 15 months now.
39 Y/O F with metastatic LCNEC of lung. Positive for following mutations; STK11, AURKA, AXL, MYC, CCNE1, GNAS, KEAP1, MCL1, RUNX1, TP53. High tumor mutation burden and PD-L1 positive.	Carboplatin and etoposide X 5 cycles. Radiological disease progression. Switched to nivolumab based on molecular tumor board recommendation.	Currently on nivolumab q 2 weeks Status post 15 doses	Radiological and clinically stable disease.

## DISCUSSION

In 2016, Rekhtman *et al*. described genomic alterations sequenced in pulmonary LCNEC and, interestingly, LCNEC patients can be subdivided into SCLC and non-SCLC (NSCLC) cohorts based on the genetic signatures of their tumor [6]. This finding implies that treating all LCNEC patients with SCLC regimens might be suboptimal. Immune checkpoint inhibition is a gratifying treatment option especially for NSCLC and could be explored for LCNEC. About 60% of pulmonary LCNEC do not exhibit the small cell hallmark signature (TP53 and Rb1 co-mutation) which might explain the large percentage of LCNEC patients who are platinum-refractory or rapidly progress on a platinum doublet. Prospective data regarding use of immune checkpoint in LCNEC is lacking but small pre-clinical data sets support further exploration of immune checkpoint in LCNEC.

Fan *et al*. studied PDL and PD-L1 expression in pulmonary neuroendocrine tumors. Ten out of 80 patients in their cohort were LCNEC. All 10 LCNEC were positive for PD-L1 and 8 out of 10 were positive for PD-1 [4]. More recently, Tsuruoka *et al*. analyzed PD-L1 expression in 227 pulmonary neuroendocrine tumors, 106 of which were LCNEC. Unlike the previous study, PD-L1 expression was modest (10.4%). Karim *et al*, recently reported PD-L1 tumoral expression in 5/24 (21%) cases albeit 2 cases with only 1% staining in 1 out of the 3 cores from each patient on the tissue microarray [7]. The variability in percentages noted in these studies may be explained by the relatively small sample numbers of LCNEC cases employed. However, in comparison to SCLC and low grade neuroendocrine tumors where 5.8% and 0% of the cases respectively were PDL-1 positive, LCNEC still exhibit a higher positivity among all pulmonary neuroendocrine tumors [5].

Although the correlation of PD-1 and PD-L1 expression with response to immune checkpoint inhibitor therapy remains under investigation, the presence of PD-1/PD-L1 in LCNEC is interesting, especially considering the scarcity of treatment options and potential therapeutic targets in this rare and very aggressive malignancy.

Clinical trials in rare tumors are difficult to conduct, hence strong prospective data regarding management of LCNEC is lacking. No prospective data regarding the use of immune checkpoint inhibitor is currently available, but is warranted.
